# Elastomeric Electrospun Scaffolds of a Biodegradable Aliphatic Copolyester Containing PEG-Like Sequences for Dynamic Culture of Human Endothelial Cells

**DOI:** 10.3390/biom10121620

**Published:** 2020-11-30

**Authors:** Luca Fusaro, Chiara Gualandi, Diego Antonioli, Michelina Soccio, Anna Liguori, Michele Laus, Nadia Lotti, Francesca Boccafoschi, Maria Letizia Focarete

**Affiliations:** 1 Tissuegraft s.r.l., 28100 Novara, Italy; luca.fusaro@med.uniupo.it; 2 Department of Chemistry “Giacomo Ciamician” and INSTM UdR of Bologna, University of Bologna, 40126 Bologna, Italy; c.gualandi@unibo.it (C.G.); anna.liguori@unibo.it (A.L.); 3 Interdepartmental Center for Industrial Research on Advanced Applications in Mechanical Engineering and Materials Technology, CIRI-MAM, University of Bologna, 40136 Bologna, Italy; 4 Department of Science and Technological Innovation and INSTM UdR Alessandria, University of Piemonte Orientale, 15121 Alessandria, Italy; diego.antonioli@uniupo.it (D.A.); michele.laus@uniupo.it (M.L.); 5 Department of Civil, Chemical, Environmental and Materials Engineering, University of Bologna, 40131 Bologna, Italy; m.soccio@unibo.it (M.S.); nadia.lotti@unibo.it (N.L.); 6 Department of Health Sciences, University of Piemonte Orientale, 28100 Novara, Italy; 7 Health Sciences & Technologies (HST) CIRI, University of Bologna, 40064 Ozzano dell’Emilia, Italy

**Keywords:** poly(butylene-co-triethylene *trans*-1,4-cyclohexanedicarboxylate), electrospinning, mechanical characterization, hemocompatibility assay, endothelial cells, elastomeric scaffold, artificial prosthesis, vascular tissue engineering, dynamic cell culture

## Abstract

In the field of artificial prostheses for damaged vessel replacement, polymeric scaffolds showing the right combination of mechanical performance, biocompatibility, and biodegradability are still demanded. In the present work, poly(butylene-co-triethylene *trans*-1,4-cyclohexanedicarboxylate), a biodegradable random aliphatic copolyester, has been synthesized and electrospun in form of aligned and random fibers properly designed for vascular applications. The obtained materials were analyzed through tensile and dynamic-mechanical tests, the latter performed under conditions simulating the mechanical contraction of vascular tissue. Furthermore, the in vitro biological characterization, in terms of hemocompatibility and cytocompatibility in static and dynamic conditions, was also carried out. The mechanical properties of the investigated scaffolds fit within the range of physiological properties for medium- and small-caliber blood vessels, and the aligned scaffolds displayed a strain-stiffening behavior typical of the blood vessels. Furthermore, all the produced scaffolds showed constant storage and loss moduli in the investigated timeframe (24 h), demonstrating the stability of the scaffolds under the applied conditions of mechanical deformation. The biological characterization highlighted that the mats showed high hemocompatibility and low probability of thrombus formation; finally, the cytocompatibility tests demonstrated that cyclic stretch of electrospun fibers increased endothelial cell activity and proliferation, in particular on aligned scaffolds.

## 1. Introduction

Cardiovascular diseases are a group of disorders of the heart and blood vessels and include coronary heart, cerebrovascular, rheumatic heart diseases, and other conditions. One of the consequences of these disabilities is the necessity to replace a damaged vessel with an artificial prosthesis. Over the years, many polymers of synthetic and natural origin have been tested for this specific application; however, the identification of polymeric materials showing the right combination of mechanical performance, biocompatibility, and biodegradability is still an open challenge [1]. The primary requirement to be satisfied is the minimization of the risk of rejection or failure in order to avoid inflammatory responses [2,3]. The ideal polymeric scaffold should show a degradation rate matching the tissue regeneration rate [1,4] and mechanical properties enabling to support the tissue formation during its degradation process [5]. In this frame, it is worth mentioning that when the degradation occurs, the chemical, physical, mechanical properties of the scaffold can significantly change, requiring therefore a careful choice of the material also on the basis of the selected strategy for the tissue formation [1]. Going into the detail of the mechanical properties to be met, in addition to support cells adhesion, growth and proliferation, a vascular scaffold should be able to withstand physiological hemodynamic forces, to show an elastomeric behavior, and to satisfy the requirement of compliance matching with the vascular tissue in order to avoid undesired consequences (i.e., anastomotic geometry, microscopic flow separation, stagnant zones) [4].

Besides the biodegradability and the mechanical properties, the biocompatibility of the scaffold plays an essential role in vascular tissue regeneration. Natural polymers intrinsically possess biological properties, such as bioactivity, susceptibility to cell-triggered proteolytic degradation, and natural remodeling, which would make them suitable for the fabrication of vascular substituents. However, these polymers are generally affected by several drawbacks, such as the potential to elicit a strong immunogenic response, the complexities associated with their purification, and the possibility of disease transmission. Among the natural polymers, collagen and elastin are the two more abundant proteins in the blood vessels; the former confers to the vessel resistance to rupture, while the latter confers elasticity [6]. Collagen is characterized by a mild-immunogenicity, showing an immune response which depends on the species from which collagen has been isolated, processing technique, and the implantation site [7]; in the field of vascular tissue engineering, collagen scaffolds have found limited applications due to their poor mechanical properties [1]. Natural elastin is a highly crosslinked insoluble polymer, and it has been observed to elicit an immune response to the same extent as collagen implants [1]. Collagen/elastin (1:1) meshes were produced by Buttafoco et al. through electrospinning of an aqueous solution of commercial soluble collagen and soluble elastin, however, for this kind of scaffold the chemical crosslinking turned out to be a mandatory step to stabilize their structure [8].

Notably, synthetic biomaterials, even though lacking specific binding sites for cell interaction, offer the opportunity to tailor mechanical properties and degradation rates. Furthermore, commercially biodegradable thermoplastic materials typically employed in tissue engineering, such as poly(lactic acid), poly(glycolic acid), polycaprolactone and their copolymers, are characterized by elastic moduli in the range 0.2–10 GPa, significantly higher than the one of native vessels (around 1 MPa) [9]. In light of these considerations, synthetic materials showing elastomeric mechanical properties, controllable degradation rate, and biocompatibility are strongly required.

In the last ten years, some of the authors have synthesized new synthetic aliphatic polyesters, properly thought for tissue engineering applications, most of which conceived as chemical modification of poly(butylene succinate) (PBS), widely employed in the biomedical field [10]. The chemical modification generally consisted in the insertion of heteroatoms (oxygen or sulfur) both in the diacid and glycol subunits. Random as well as block copolymers of PBS containing a diglycolic acid subunit turned out to be suitable for bone tissue engineering [11,12]. The heteroatoms were also inserted in the glycol subunit: such chemical modification conferred flexibility and increased hydrophilicity to the resulting scaffolds [13,14]. Random and block copolymers of PBS containing PEG-like moieties, characterized by remarkable hydrophilicity and flexibility, were also synthesized [15,16]. Another interesting aliphatic polyester, which is gaining importance for its potential use in biomedical applications, due to its proven biocompatibility, high chemico-thermal stability and very good mechanical properties, is poly(butylene *trans*-1,4-cyclohexanedicarboxylate) (PBCE). Such polyester is characterized by the presence of a 6 carbon atom cycloaliphatic ring per repeat unit. In a previous work, its chemical structure was ad hoc modified by random insertion along the macromolecular chain of different amounts of diglycolic acid. The resulting new polymer was employed for the obtainment of very flexible and fast degrading films, showing good biocompatibility as indicated by preliminary tests [17]. The authors also synthesized a series of random copolymers of PBCE containing different amount of triethylene glycol moieties in order to explore their suitability to form compostable films for food packaging. The mechanical response and the hydrophilicity of PBCE resulted markedly changed by copolymerization and strongly affected by the amount of PEG-like sequences introduced: the higher the amount of the comonomeric units the higher the final hydrophilicity and flexibility [18]. From a couple of these copolymers, electrospun scaffolds showing aligned nanofibers properly designed for skeletal muscle tissue regeneration were prepared [19]. The results highlighted the influence of chemical composition, and, in particular, of ether linkages, on scaffold’s mechanical properties, hydrolytic degradation rate, and density of cell anchoring points. Furthermore, the fibrous morphology of the scaffold clearly favored cell alignment along the fiber direction and allowed cell infiltration and oxygen and nutrient diffusion [19].

Among the techniques proposed for the fabrication of scaffolds for vascular tissue engineering [20], the electrospinning technique, offering precise control over the dimension and alignment of fibers, enables to properly tune the structural and mechanical properties as required for the specific application [21]. Moreover, electrospun aligned nanofibers allow orienting cells in a specific direction, mimicking the anisotropy encountered in certain organs, including blood vessels [21]. In the past few years, different approaches have been proposed for the fabrication of scaffolds for blood vessels by means of the electrospinning technique. Niu et al. produced bioresorbable electrospun tubular scaffolds of poly(L-lactide-co-caprolactone) with randomly, circumferentially and axially aligned structures, showing a nonlinear elasticity comparable to the ones of arteries in the human body and an alignment of the cells, when seeded on axially aligned fibers, in the direction of the fibers [22]. This result is in line with the outcome of the previous work of Xu et al., in which human coronary artery smooth muscle cells seeded on aligned poly(L-lactide-co-caprolactone) nanofibrous scaffold proliferated along the longitudinal direction of the nanofibers, forming a pattern similar to the one of the native arteries [23]. In order to improve the antithrombogenic properties of polycaprolactone (PCL) electrospun scaffolds for vascular tissue engineering, an oxygen plasma treatment of the mat, aimed at inducing the graft copolymerization of acrylamide monomer, has been recently proposed [24]. In another very recent study, small diameter vascular grafts were produced from electrospun polyurethane/PCL mats that were chemically grafted with linoleic acid, an antithrombogenic fatty acid in order to prevent blood coagulation without affecting the mechanical properties [25]. Electrospun vascular graft scaffolds were also produced starting from blends of synthetic and natural polymers. Aydogdu et al. have recently proposed the use of PCL, ethyl cellulose and collagen type-1 to obtain electrospun scaffolds for the mimicking of native small diameter blood vessels [26]. Electrospun PCL/chitosan and PCL/gelatin scaffolds were also tested, turning out to be biocompatible and able to support cell attachment, growth and long-term proliferation [27,28].

In the present work, poly(butylene-co-triethylene *trans*-1,4-cyclohexanedicarboxylate) (P(BCE-co-TECE)), a biodegradable random aliphatic copolyester of butylene cyclohexanedicarboxylate containing 30 mol % of triethylene glycol moieties, was synthesized and processed to obtain electrospun scaffolds properly designed for vascular applications. Scaffolds characterized by both random and aligned fibers were subjected to mechanical characterization carried out through tensile and dynamic-mechanical tests, these last performed under conditions that simulate the mechanical contraction of vascular tissue in terms of wall elongation and pulse frequency. Lastly, to evaluate the potential use of the so-prepared scaffolds for vascular tissue engineering, biological tests were carried out, i.e., hemocompatibility and MTS assays.

## 2. Materials and Methods

### 2.1. Materials

*Trans*-1,4-cyclohexanedicarboxylic acid (CEDA) (trans 99 mol%) was purchased from Zentek (partner of TCI, Milan, Italy), while all the other reagents used were obtained by Sigma Aldrich (Milan, Italy). CEDA, 1,4-butanediol (BD), triethylene glycol (TEG), 2,2,2-Trifluoroethanol (TFE), N,N-Dimethylacetamide and titanium tetrabutoxide (Ti(OBu)_4_) were reagent-grade products; all reagents were used as supplied.

### 2.2. Polymer Synthesis

P(BCE-co-TECE) copolymer was prepared by two-stage melt polycondensation, as previously reported [18]. Specifically, we start from CEDA and BD/TEG molar ratio of 70/30. To favor the shift of reaction towards products, a 20 mol% excess of the glycol mixture with respect to CEDA was used. Ti(OBu)_4_, employed as catalyst, was added in a quantity of 150 ppm. The synthesis was carried out in a 250 mL stirred glass reactor in a thermostated silicon oil bath with continuous recording of temperature and torque values. In the first step, conducted under nitrogen flow, the temperature was kept at 180 °C. These conditions were maintained until the theoretical amount of water was distilled off (about 90 min). In the second stage, the pressure was progressively reduced to 0.1 mbar, and the temperature risen to 220 °C, to remove the glycol excess, thus favoring the molecular weight growth; such step was extended for a period of three hours, the time needed to reach out a constant torque value.

### 2.3. Preparation of Electrospun Scaffolds

Electrospun scaffolds were produced by using an in-house electrospinning apparatus composed of a high voltage power supply (Spellman, Hauppage, NY, USA, SL 50 P 10/CE/230), a syringe pump (KDScientific 200 series, Holliston, MA, USA), a glass syringe, a stainless-steel blunt-ended needle (inner diameter = 0.51 mm) connected with the power supply electrode and a grounded rotating collector (length = 120 mm, diameter = 50 mm, rotational speed up to 16.2 m/s). The polymer solution was dispensed, through a Teflon tube, to the needle vertically placed on the collecting mandrel. The electrospinning process was carried out at room temperature (RT) and relative humidity RH = 40–50%. The polymer was dissolved in TFE at a concentration of 22% *w*/*v*. The polymeric solution was electrospun by applying the following processing conditions: applied voltage = 22 kV, feed rate = 0.6 mL/h, needle-to-collector distance = 22 cm. Fibers were collected with a random arrangement on the cylinder rotating at a speed of 5.2 m/s, whereas aligned fibers were obtained by increasing the rotational speed up to 16.2 m/s. Scaffolds with random and aligned fibers were labelled “RND” and “ALN” respectively.

### 2.4. Characterization Methods

Chemical structure was confirmed, while composition and *trans/cis* isomer ratio was estimated by ^1^H-NMR spectroscopy and the degree of randomness b was calculated from ^13^C-NMR analysis.

The polymer was dissolved (30 mg/mL) in chloroform-d solvent with 0.03 vol.% tetramethylsilane added as an internal standard. The measurements were carried out at RT, employing a Varian INOVA 400 MHz instrument.

Molecular weight data were obtained by gel-permeation chromatography (GPC) at 30 °C using a 1100 Hewlett Packard system equipped with PL gel 5µ MiniMIX-C column (250/4.6 length/i.d., in mm). A refractive index was employed as detector. In all cases, chloroform was used as eluent with a 0.3 mL/min flow and sample concentrations of about 2 mg/mL. A molecular weight calibration curve was obtained with polystyrene standards in the range of molecular weight 2000–100,000 g/mol.

Differential scanning calorimetry (DSC) measurements were carried out using a TA Instruments Q100 DSC equipped with the refrigerator cooling system accessory. DSC scans were performed from −90 °C to 200 °C in nitrogen atmosphere. A rate of 20 °C/min was used during heating scans, whereas the cooling scans were performed at a rate of 10 °C/min. The glass transition temperature (T_g_) was taken at half-height of the glass transition heat capacity step whereas the melting temperature (T_m_) was taken at the peak maximum of melting endotherm. The degree of crystallinity (*χ_c_*) was calculated by using Equation (1):

(1) $$\chi_{c}=\frac{\Delta H_{m}}{\Delta H_{m}^{0}}\cdot 100$$

where Δ*H_m_* is the malting enthalpy associated to the first heating scan, and $\Delta H_{m}^{0}$ is the theoretical melting enthalpy of the 100% crystalline PBCE homopolymer, equal to 78 J/g [29].

Scanning electron microscope (SEM) observations were carried out using a Leica Cambridge Stereoscan 360 scanning electron microscope at an accelerating voltage of 20 kV, on samples sputter-coated with gold. The distribution of fiber diameters was determined through the measurement of about 200 fibers by means of an acquisition and image analysis software (ImageJ), and the results were given as the average diameter ± standard deviation. Fiber orientation was estimated by elaborating SEM images with ImageJ by applying the directionality plug-in, which exploits the local gradients orientation method. Four images were analyzed to determine the fraction of fibers oriented in a certain direction (with 90° corresponding to the direction of drum circumference), and average values were reported in a histogram with corresponding standard deviations.

Stress–strain measurements were performed with an Instron 4465 tensile testing machine on rectangular sheets cut from electrospun mats. Sample was 5 mm wide, the gauge length was 20 mm and the cross-head speed was 5 mm/min. Thickness was measured for each specimen by using a microcaliper. The ALN scaffold was tensioned in the direction of fiber axes. Load-displacement curves were obtained and converted to stress strain curves. At least six replicate specimens were run for each sample and results were provided as the average value ± standard deviation.

Dynamic-mechanical tests were performed using a dynamic-mechanical analyzer DMTAV (Rheometric Scientific, New Castle, DE, USA) in inverted position employing rectangular tension geometry. A time sweep analysis was carried out for 24 h while the sample was immersed in a bath with phosphate buffer solution at 37 °C. A strain amplitude of 5% and a deformation frequency of 1 Hz were chosen.

### 2.5. Hemocompatibility Assay

Blood interaction with electrospun scaffolds was evaluated with thromboelastography (TEG). Blood samples were collected in Vacutainer (BD, Franklin Lakes, NJ, USA) containing sodium citrate. Blood was put in contact to electrospun samples for 30 min at 37 °C and analyzed using Thromboelastograph^®^ (TEG^®^ 5000 Thrombelastograph^®^ Hemostasis Analyzer System) and the Kaolin TEG standard protocol. Briefly, after 30 min 1 mL of blood was collected and placed in a vial containing kaolin. After few seconds, 340 µL of blood were sampled in the thrombelastograph with 20 µL of 200 mM calcium chloride, in order to eliminate the anticoagulant effect of sodium citrate. After the test, the machine displayed a thromboelastogram, from which coagulation parameters were obtained, such as reaction time, coagulation time, platelet activation and fibrinogenic activity.

### 2.6. Cell Culture

Human endothelial EA.hy926 cells (ATCC CRL-2922, Manassas, VA, USA) were cultured in high-glucose Dulbecco’s modified Eagle’s medium (DMEM) with 10% fetal bovine serum, penicillin (100 U/mL), streptomycin (100 µg/mL), and 2 mM glutamine mixture (all from Euroclone, Milan, Italy), at 37 °C with 5% CO_2_ and 80% of relative humidity. In order to test cell behavior on electrospun scaffolds, samples of 3 × 2 cm were cut, disinfected in a solution of 70% Ethanol (Sigma-Aldrich, Milan, Italy) and placed in a TC-3 bioreactor (EBERS Medical Technology, Zaragoza, Spain). Fifty thousand cells were seeded on each sample. After 24 h, during which cells adhered to the scaffold’s surface, samples underwent a cyclic stretch of 5% of elongation and frequency 1 Hz. At the same time, cells were cultured on electrospun samples of the same size and maintained in static conditions. After three and seven days, samples were collected for further analysis. Experiments were performed in triplicate.

### 2.7. Viability Test

In order to evaluate cell viability, the MTS assay (CellTiter 96^®^ aqueous nonradioactive cell proliferation assay, Promega, Italy) was performed, following instructions provided by the manufacturer. Samples cultured under static and dynamic conditions were placed in a six-well plate and a 3-(4,5-dimethylthiazol-2-yl)-5-(3-carboxymethoxyphenyl)-2-(4-sulfophenyl)-2H-tetrazolium solution in DMEM was added and incubated for 4 h. Subsequently, 100 µl of medium for each sample were analyzed by UV-VIS spectrophotometry (V-630 UV-Vis Spetrophotometer, Jasco, Oklahoma City, OK, USA), at a wavelength of 490 nm. Absorbance measures were proportional to cell viability.

### 2.8. Fluorescence Assay

To assess cell morphology on electrospun samples, phalloidin staining was performed. Collected samples were fixed with a solution of 4% formalin for 2 h at RT, then incubated with phalloidin-tetramethylrhodamine conjugated (TRITC) (Sigma Aldrich, Milan, Italy) for 45 min at 37 °C. 4′,6-Diamidine-2′-phenylindole dihydrochloride (DAPI Sigma Aldrich, Milan, Italy) was used for nuclear staining. Samples were observed at fluorescent microscope (DM2500 Leica, Wetzlar, Germany).

### 2.9. Statistical Analyses

The experiments of hemocompatibility and cell viability were repeated at least three times. All results are reported as mean values ± standard deviation. Student’s *t*-test was performed to assess the statistical significance. * indicates *p* ≤ 0.05.

## 3. Results and Discussion

**Figure 1:**
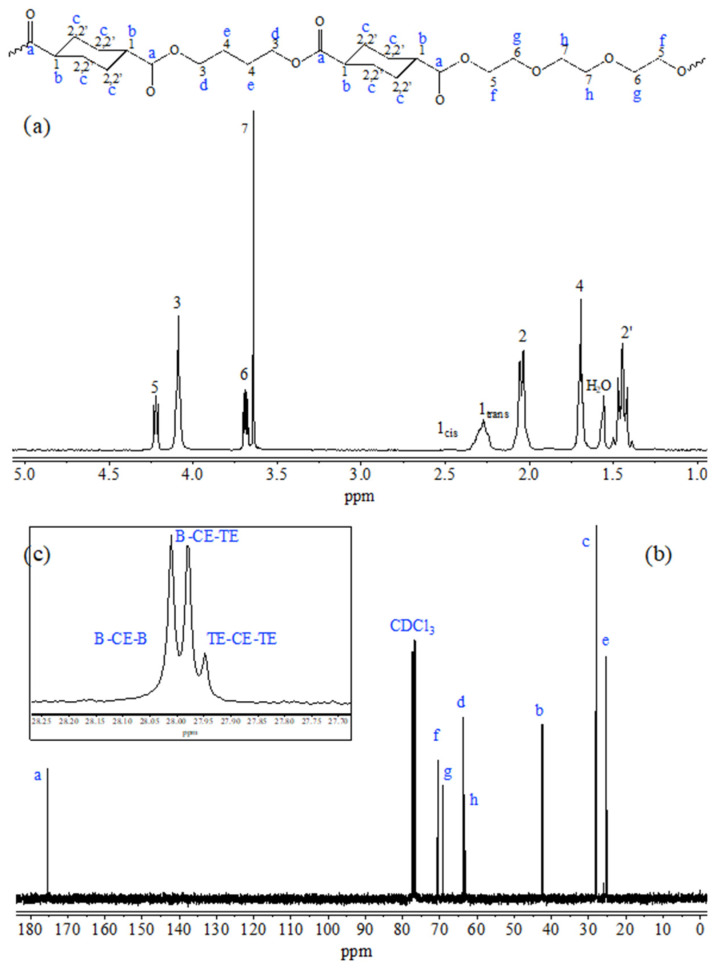
^1^H-NMR (**a**) and ^13^C-NMR (**b**) spectra of P(BCE-*co*-TECE) copolymer with the relative peak assignment. Insert (**c**): magnification of the 28.25–27.70 ppm region.

As evidenced by the chemical formula reported in Figure 1, the copolymer contains a PEG-like moiety along the macromolecular chain, characterized by the presence of two ether oxygen atoms regularly distributed within a highly flexible aliphatic glycol subunit composed of six carbon atoms (one oxygen for every two methylene groups). As is well known from the literature [10,12], the presence of polar ether linkages favors the initial cell attachment and, at the same time, makes the material less rigid. The particular composition of the copolymer guarantees the proper balance between degree of crystallinity, necessary to allow sample processability through electrospinning, and appropriate mechanical properties (copolymer elastic modulus is 1/3, whereas copolymer elongation at break is 12 times higher than those of PBCE homopolymer) [18].

Figure 1 reports the chemical structure (top), the ^1^H-NMR (a) and ^13^C-NMR (b and c) spectra of P(BCE-co-TECE) copolymer, with the relative peak assignment (numbers for H atoms and letters for C ones). Both NMR spectra contain just the signals due to the atoms of copolymer structure, allowing to exclude the occurrence of side reactions during polymerization process. The actual composition was calculated from the normalized area of the peaks 3 and 5 ascribable to butylene and triethylene moieties, respectively. The molar amount of BCE and TECE co-units are 71 and 29 mol%, respectively, very close to the feed one. From the relative intensity of the 1*trans* and 1*cis* peaks, due to the *trans* and *cis* isomers respectively, a 2 mol% of *cis* isomer was calculated, proving that the polymerization conditions permit to avoid isomerization side reactions usually occurring at high temperatures.

The degree of randomness *b* was determined from the peaks located at 28 ppm (insert c) where the c carbons of the aliphatic ring are located. As can be seen, three different signals for these carbon atoms can be detected: the peak at 28.02 ppm corresponding to *B-CE-B* triads; the signal at 27.98 ppm due to *B-CE-TE* triads; the peak at 27.95 ppm related to the *TE-CE-TE* triads; *B*, *TE* and *CE* representing butylene, triethylene and cyclohexane moieties, respectively. In particular, *b* was calculated according to the equation:

$$b=P_{B-TE}+P_{TE-B}$$

where *P_B-TE_* and *P_TE-B_* are the probability of finding a *B* glycol subunit next to a *TE* one and the probability of finding a TE moiety followed by a *B* one, respectively, and can be expressed as follows:

PB−TE=IB−CE−TE2IB−CE−TE2+IB−CE−B; PTE−B=IB−CE−TE2IB−CE−TE2+ITE−CE−TE

where *I_B-CE-B_*, *I_B-CE-TE_*, *I_TE-CE-TE_* representing the normalized intensities of the resonance peaks of the *B-CE-B*, *B-CE-TE*, *TE-CE-TE* triads, are 1.22, 1 and 0.2, respectively. The value of *b* thus calculated is 1, indicating the random distribution of the different counits along the macromolecular chain.

The molecular characterization was completed by GPC analysis that allowed determining the number molecular weight (M_n_) and the polydispersity index (D), 31,000 Da and 2.2, respectively. The high M_n_ value, together with D parameter close to 2 and a very low *cis* isomer content, prove a good control of polymerization process.

**Figure 2:**
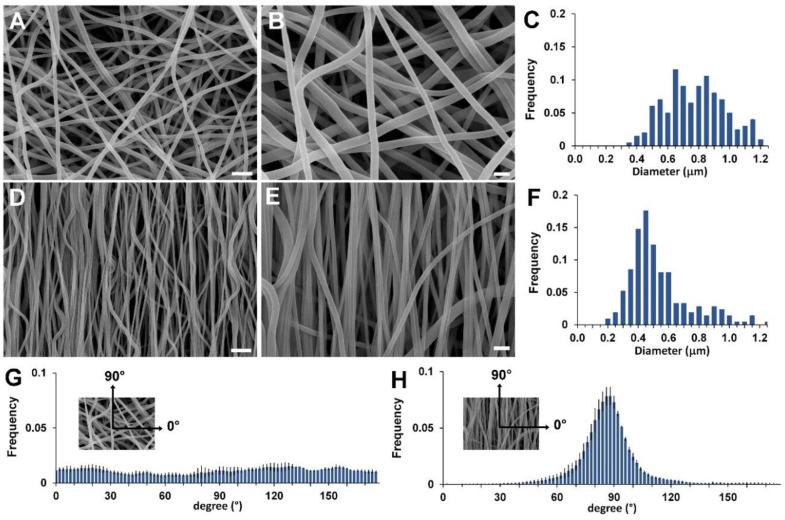
SEM images of RND scaffold (**A**,**B**) and ALN scaffold (**D**,**E**). Fiber diameter distribution of RND scaffold (**C**) and ALN scaffold (**F**). Fiber orientation distribution of RND scaffold (**G**) and ALN scaffold (**H**). Scale bars: A and D = 5 µm; B and E = 2 µm.

The electrospinning conditions employed to fabricate the scaffolds are the result of a set of experiments aimed at optimizing the electrospinning parameters to obtain beadfree submicrometric fibers, as shown in Figure 2A,B,D,E for the RND and the ALN scaffold, respectively. The two scaffolds slightly differ in the fiber diameter distribution (Figure 2C,F, 800 ± 190 nm vs. 560 ± 210 nm, for the RND and the ALN scaffold, respectively), as a consequence of mechanical drawing that reduces the diameter of the fibers when collected on the high rotating drum [30,31]. Mostly, the two scaffolds differ in fiber orientation, as clearly shown by SEM images and by the quantitative analysis of fiber orientation distribution (Figure 2G,H), that demonstrates the preferential orientation of fiber axis in the direction of drum circumference for the ANL scaffold and the fiber random orientation in the case of RND scaffold.

**Figure 3:**
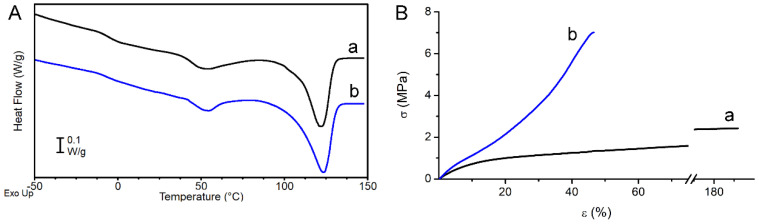
(**A**) DSC first heating scans of RND (a, black) and ALN (b, blue) scaffolds. (**B**) Stress–strain curves of RND (a, black) and ALN (b, blue) scaffolds.

Sample thermal properties were investigated by DSC analysis. Figure 3A reports the DSC curves and Table 1 the obtained calorimetric data referred to the first heating scan of RND and ALN scaffolds. The two scaffolds display similar DSC curves: the polymer is semicrystalline with a T_g_ below RT and a crystal phase that melts in a broad temperature range (40–135 °C) with multiple melting peaks. The latter are ascribable to melt and recrystallization processes typically occurring during the DSC scan in polyesters, especially in the presence of a large distribution of crystallites with different degree of perfection [32]. As previously reported, in this type of random copolymers, BCE units crystallize whereas the TECE counits are completely rejected from the crystal lattice of BCE, but increase the overall disorder in the molecular structure, thus decreasing the crystallinity degree when compared to the PBCE homopolymer [18]. From an application perspective, the most important consequence of this structure-correlated phenomenon is the decrease of polymer elastic modulus, being this kind of copolymer characterized by a soft amorphous phase (T_g_ < RT) [18].

Tensile mechanical properties of the investigated polymers are reported in Table 1, where elastic modulus E, stress at break σ_b_, and deformation at break ε_b_ are listed. Figure 3B shows a representative stress–strain curve of each sample. The RND scaffold has a lower elastic modulus, lower stress at break and higher deformation at break compared to the ALN scaffold. SEM analysis highlighted that the two scaffolds differ in fiber diameter and fiber orientation. It was extensively demonstrated that the fiber diameter has a remarkable effect on the elastic modulus at the single fiber level [33,34,35,36], whereas the overall mechanical properties of the nonwoven structure were found to be mostly affected by the arrangement of the fibers, such as fibers direction, fibers curvature and fibers fusion at contact points [37,38,39,40]

In the present work, as a matter of fact, the different mechanical properties of the two scaffolds are ascribable to the different fiber orientation and can be explained by considering that the contribution of each individual fiber to the overall mat mechanical behavior depends on its orientation with respect to the loading direction. In the ALN scaffold most of the fibers are parallel to the loading direction and contribute to load bearing, leading to a higher elastic modulus compared to RND scaffold. In the latter, instead, most of the fibers experience a change of orientation upon loading to be engaged for bearing the load [37]. Therefore, fiber alignment has the effect of strengthening the scaffold (ALN sample has higher elastic modulus and higher stress at break than RND sample), but reduces scaffold flexibility (ε_b_ is 44% and 176% for ALN and RND scaffold, respectively).

The mechanical properties of the investigated scaffolds seem to fit within the range of physiological properties for medium and small caliber blood vessels [41,42]. It is also pointed out that the ALN scaffold displays a strain-stiffening behavior typical of the blood vessels [43].

**Figure 4:**
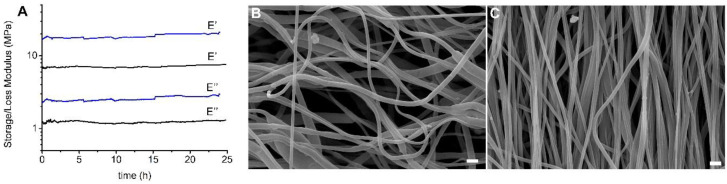
(**A**) Dynamic-mechanical curves of RND (black) and ALN (blue) scaffolds. SEM images after dynamic-mechanical analysis of RND (**B**) and ALN (**C**) scaffolds. Scale bar = 2 µm.

Dynamic-mechanical analysis was carried out to evaluate the mechanical performance of the scaffolds under conditions that simulate the mechanical contraction of vascular tissue in terms of wall elongation and pulse frequency [41]. Tests were carried out by immersing the specimens in PBS solution at 37 °C for 24 h and applying a strain amplitude and deformation frequency of 5% and 1Hz, respectively. In Figure 4A trends of storage modulus E’ and loss modulus E’’ are reported as a function of time for RND and ALN scaffolds. As expected, and in line with mechanical data determined by stress–strain tests on dried samples, the ALN scaffold shows higher moduli than the RND one. For both scaffolds, storage and loss moduli are constant in the timeframe investigated, demonstrating the stability of the scaffolds under the applied conditions of mechanical deformation. The slight increase of E’ of ALN scaffold can be related to a gradual slippage of the sample from the grip. Further confirmation of the structural stability of the scaffolds is provided by SEM images (Figure 4B,C) acquired after the dynamic-mechanical test, that highlight that the morphology of both materials remained unaltered.

Wettability and hydrolytic degradation of P(BCE-co-TECE) electrospun scaffolds, which are important properties in view of tissue engineering application, have been previously investigated by some of the authors. It was demonstrated that the presence of oxygen-containing functional groups increases the polar component and hydrophilicity of the polymer surface, contributing to increase the surface wettability [19]. Moreover, the hydrolysis profile of P(BCE-co-TECE) has been investigated in physiological environment [19] and results showed that in a time interval between few days and seven months, the polymer underwent a decrease of number average molecular weight with time of about 30%, but it maintained integrity and no significant weight loss was measured [19].

**Figure 5:**
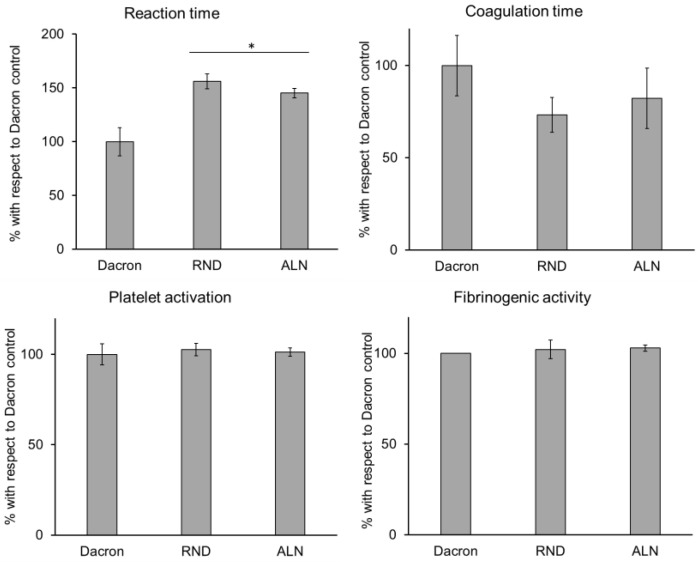
Hemocompatibility test on coagulation parameters evaluated on synthetic vascular substitutes (Dacron), RND, and ALN scaffolds. Data are expressed as mean values ± SD. (*n* = 3 from different healthy donors, * *p* ≤ 0.05).

In order to verify electrospun samples’ compatibility to vascular tissue, several biological parameters were considered. At first, hemocompatibility was assessed, and the behaviour of blood in contact to RND and ALN was evaluated. As widely used in vascular substitution, results were compared to a Dacron control. As showed in Figure 5, all parameters considered for electrospun samples showed no significant difference compared to Dacron control, except for reaction time, which is prolonged on RND and ALN scaffolds. Reaction time represents the time needed for the beginning of the coagulation process, and, while this parameter for electrospun scaffolds still fits the range of physiological values [44], an increased reaction time could imply a decrease of probability of thrombus formation, a common problem in vascular substitution [45,46].

To evaluate the behavior of endothelial cells seeded onto electrospun scaffolds, and therefore scaffolds’ cytocompatibility with cells of vascular environment, EA.hy926 viability and morphology were assessed. Concerning viability, results showed that after three days of culture there is no significant difference between RND and ALN both in static and dynamic conditions, while all the samples’ viability results significantly lower than the viability on plastic control.

**Figure 6:**
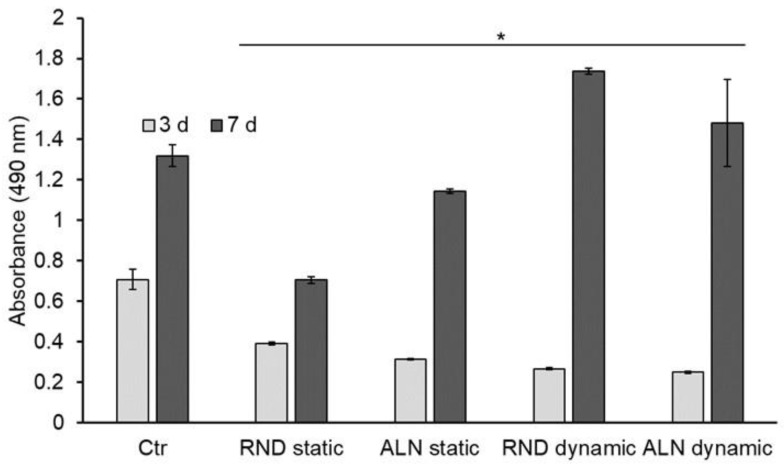
Endothelial cells viability assay. MTS assay was performed after three and 7seven days of culture on static and dynamic samples. Data are expressed as mean values ± SD. (*n* = 3, * *p* ≤ 0.05 with respect to control).

After seven days, cell viability is significantly higher on dynamically stretched samples compared with the static, while no differences were displayed between RND and ALN samples. Thus, dynamic conditions seemed to improve cell culture conditions (Figure 6).

**Figure 7:**
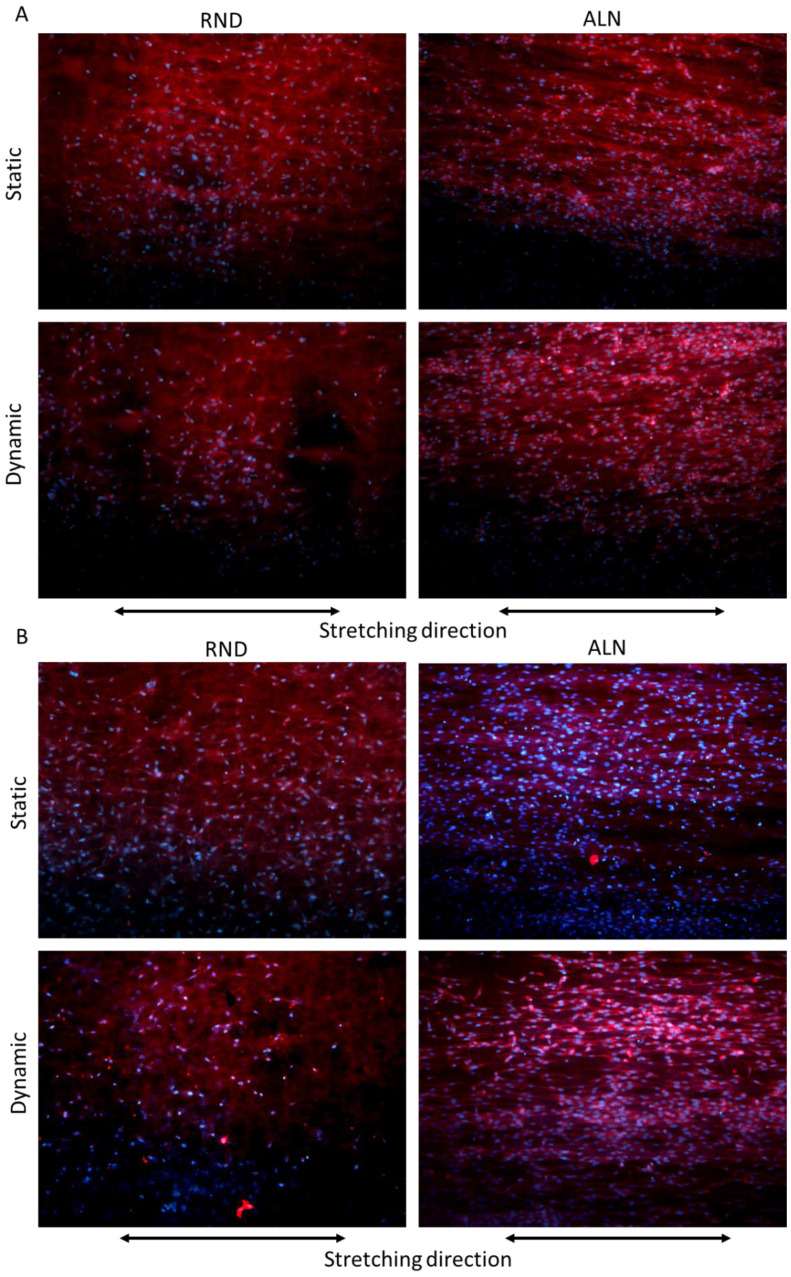
Phalloidin staining of endothelial cells cultured on RND and ALN scaffolds for three (**A**) and seven days (**B**). Pictures are representative of three different experiments.

Cell morphology, evaluated through phalloidin fluorescence assay after three days, confirmed the viability assay, showing no significant difference between the two scaffolds in terms of cell adhered, while cell morphology on ALN samples is guided by both fibers alignment and stretching direction, highlighting a significant alignment of cells on dynamic ALN sample (Figure 7A). On the other hand, after seven days, cells proliferate on ALN samples both in static and dynamic conditions, and their morphology adapted more efficiently to fiber and stretching direction, as evidenced in particular on dynamically stretched samples (Figure 7B). Overall, cytocompatibility seemed to indicate that cyclic stretch on electrospun fibers increased endothelial cell activity and proliferation, in particular on ALN samples. Fast proliferation of endothelial cells could represent an advantage in vascular applications. Indeed, in blood vessels, the inner layer in contact with blood flow, called tunica intima, is composed of endothelial cells and possess anticoagulant and angiogenetic properties [47,48]. In this case, results seem to indicate an unusual affinity of endothelial cells for electrospun scaffold, unlike other compounds normally used in vascular surgery, which always showed poor endothelial cell recruitment properties and has always represented a big drawback for synthetic materials [49].

## 4. Conclusions

Random and aligned electrospun P(BCE-*co*-TECE) scaffolds properly thought for damaged vessel replacement were realized and characterized. P(BCE-*co*-TECE), a biodegradable random aliphatic copolyester of poly(butylene *trans*-1,4-cyclohexanedicarboxylate) containing 30 mol % of triethylene glycol moieties, characterized by the presence of two ether oxygen atoms regularly distributed within a highly flexible aliphatic glycol subunit composed of six carbon atoms, was prepared by two-stage melt polycondensation. The characterization of the synthesized macromolecule confirmed a good control of the polymerization process. The DSC analysis performed on the electrospun scaffolds highlighted the crystallization of BCE units, while TECE counits were completely rejected from the crystal lattice of BCE, with a resulting decrease of the crystallinity degree with respect to the PBCE homopolymer. Concerning the mechanical properties, random scaffolds showed lower elastic modulus (9.3 MPa vs. 16 MPa), lower stress at break (2.4 MPa vs. 8 MPa) and higher flexibility (ε_b_: 176% vs. 44%) compared to the aligned scaffolds, ascribable to the fact that in the aligned scaffolds most of the fibers were parallel to the loading direction. Dynamic-mechanical analysis carried out in dynamic conditions, by immersing the specimens in PBS solution at 37 °C for 24 h and applying a strain amplitude of 5% and deformation frequency of 1Hz, confirmed the differences in elastic modulus for the two typologies of scaffolds and highlighted the stability of both random and aligned scaffolds under the applied conditions. The structural stability of the scaffolds was also reported by SEM characterization performed after dynamic-mechanical tests. The cytocompatibility analysis performed on random and aligned scaffolds indicated a relevant increase in endothelial cell activity and proliferation on aligned samples when subjected to a cyclic stretch. This result, together with the positive outcomes of hemocompatibility and mechanical characterizations, demonstrated the suitability of the proposed scaffolds for applications as artificial prosthesis for blood vessel replacement and restoration.

## Figures and Tables

**Table 1:** Calorimetric and mechanical data of RND and ALN scaffolds.

Sample	T_g_ (°C)	T_m_ (°C) ^a^	ΔH_m_ (J/g)	Χ_c_ (%) ^b^	E (MPa)	σ_b_ (MPa)	ε_b_ (%)
RND	−5	53, 122	31	40	9.3 ± 0.7	2.4 ± 0.3	176 ± 20
ALN	−3	53, 124	29	37	16 ± 2	8 ± 1	44 ± 2

^a^ Multiple melting peaks; ^b^ degree of crystallinity calculated according to Equation (1).
